# Assessing public transport accessibility for people with physical disabilities in burgos, spain: A user-centered approach to inclusive urban mobility

**DOI:** 10.1371/journal.pone.0322068

**Published:** 2025-04-29

**Authors:** Juan L. Elorduy, Yesica Pino, Ángel M. Gento

**Affiliations:** Department of Management Science, Universidad de Valladolid, Valladolid, Spain.; IULM: Libera Universita di Lingue e Comunicazione, ITALY

## Abstract

**Background:**

Public transport accessibility is vital for fostering inclusive and sustainable urban development, ensuring equitable mobility for individuals with physical disabilities or reduced mobility. Globally, over 1.3 billion people, including 4.12 million in Spain, live with disabilities. Despite legislative progress, many cities fail to meet accessibility standards that guarantee safe and independent public transport use.

**Aim:**

This study evaluates the accessibility of 431 bus stops in Burgos, Spain, using a methodological design that combines compliance with accessibility standards and the lived experiences of individuals with disabilities. By identifying critical barriers and opportunities for improvement, this research provides actionable insights for urban planners and policymakers, offering a replicable framework for cities facing similar challenges.

**Methods:**

A validated and reproducible methodology was employed to evaluate accessibility conditions through in situ observations, including the geolocation of bus stops and photographic documentation. This approach guarantees a user-centred perspective through collaboration with local disability organisations.

**Results:**

The analysis identified significant barriers, including inadequate vehicle encroachment prevention, poorly designed shelters, unsuitable stop locations, and limited accessible formats such as Braille and audio. These challenges hinder the independent use of public transport for individuals with disabilities or reduced mobility.

**Conclusions:**

Addressing these barriers can substantially enhance urban mobility, reduce environmental impacts, and support Sustainable Development Goal 11, particularly Target 11.2. The proposed methodological design provides a practical framework for urban planners to create more inclusive, resilient, and sustainable transport systems globally.

## Introduction

Transport is essential for human development, because it facilitates access to resources and opportunities [1]. It plays a key role in urban development by connecting people to activities such as employment, education, healthcare, leisure, social participation [2,3], and other related activities. According to Miralles-Guasch [4], it is inconceivable to separate the city from transport. Transport and cities are intertwined in a complex relationship involving people, needs, services, and space. The creation of an accessible and functional public transport network generates significant benefits in terms of economic activity, quality of life, environmental sustainability, and the overall success of interconnected cities [5], while simultaneously addressing critical urban challenges such as social exclusion, sustainability, and the efficient use of public space.

Sustainable Development Goals (SDGs) [5] represent a global effort to address critical challenges and promote sustainable development across multiple domains. Goal 11 focuses on sustainable cities and communities, aiming to create human settlements that are inclusive, safe, resilient, and sustainable. Specifically, Target 11.2 seeks to ‘ensure universal access to safe, affordable, accessible, and sustainable transport systems and improve road safety by 2030, paying particular attention to the needs of people in vulnerable situations, such as women, children, persons with disabilities, and older people. The implementation of these policies not only enhances mobility, but also strengthens the resilience and inclusivity of urban environments [6], aligning with the growing need for equitable transport systems.

Public transport is essential for creating sustainable cities by ensuring mobility for all, reducing greenhouse gas emissions, and improving air quality. Efficient public transport systems alleviate traffic congestion and optimise the use of urban space [7,8]. Improving accessibility to public transport and its infrastructure plays a key role in encouraging a modal shift from private vehicles to public transport, thereby reducing noise pollution, CO₂ emissions, and traffic congestion in urban areas [9].

Access to transport is a prerequisite for participation in community life [10], making it essential to ensure that all individuals can fully engage in urban activities [11,12]. Mobility limitations often lead to social exclusion [13,14], and people with disabilities have been identified as one of the most affected groups [15,16]. Difficulties in accessing public transport are an important cause of this exclusion [17,18]. Improving public transport accessibility is essential not only for people with disabilities but also for other vulnerable groups, such as older adults and pregnant women [19,20].

The Convention on the Rights of Persons with Disabilities [21] promotes the full enjoyment of human rights and fundamental freedom for persons with disabilities. Article 9 requires States to adopt measures to ensure equal access to physical environment and transportation. Similarly, the Spanish Constitution [22] calls on public authorities to implement policies that guarantee personal autonomy and social inclusion in universally accessible environments. To achieve independent living and full societal participation, governments must prioritise the elimination of transport accessibility barriers [23]. These efforts are crucial for advancing inclusive and sustainable urban mobility.

Facilitating the accessibility of transport for people with disabilities is critical, as the demand for accessible transport services is expected to increase in the future. Globally, there are 1.3 billion people with disabilities, representing about 16% of the population [24]. In Spain, it is estimated that 4.12 million people have disabilities, accounting for approximately 9% of the total population [25]. Among individuals with disabilities aged six and over, 40.33% experience difficulties using public transportation. The primary barrier cited was ‘getting on or off the vehicle or accessing a seat,’ reported by 81.75% of respondents, followed by ‘access to stations, platforms, and stops’ (67.14%). Orientation difficulties, such as navigating stations, understanding signage, maps, and routes, and identifying the correct stop, affected 54.61% of people with disabilities, while 17.44% reported other types of problems [25].

Additionally, as the population ages, the number of people facing difficulties using public transport is expected to rise. In Spain, individuals over 65 currently represent more than 20% of the population, and this figure is projected to reach 30.5% by 2055 [26]. There is a strong correlation between ageing and disability, as the incidence of disability increases significantly with age [27]. These demographic trends highlight the pressing need to develop accessible and sustainable transport systems that address the growing mobility challenges of an ageing population.

Cities face significant challenges in meeting the requirements of Sustainable Development Goal (SDG) 11, specifically target 11.2, which aims to ensure universal access to safe, affordable, accessible, and sustainable transport systems for all, with particular attention to vulnerable groups, such as persons with disabilities and older adults. The lack of accurate data on accessibility needs and insufficient coordinated governance hinders the implementation of effective solutions [28,29]. Despite legislative efforts to improve accessibility, studies indicate that urban centres continue to pose significant challenges for users with physical disabilities and/or reduced mobility [30–32]. The persistent failure to integrate the needs of people with disabilities into urban design highlights the necessity for innovative approaches and collaborative governance to address these barriers effectively [33].

Despite the increase in literature on public transport and disability, research in this area remains relatively scarce. While several authors have explored the topic, the publication frequency is low, and studies often lack interconnectedness. A limited amount of work has thoroughly addressed the current state of public transport accessibility from the perspective of people with disabilities [34–39]. However, recent studies have expanded to include the perspectives of other stakeholders, such as support staff [40], flight attendants [41], drivers [42], transport planners [43], and policymakers [44]. These studies have primarily focused on identifying the barriers faced by people with disabilities, but few have proposed comprehensive solutions to these challenges. This lack of integrative approaches limits the potential for creating sustainable and inclusive urban transport systems, which is a critical objective for contemporary urban planning.

Some studies have examined the infrastructure challenges and urban design issues that affect public transport accessibility for people with disabilities [45,46], highlighting the importance of incorporating universal design principles to improve accessibility of both infrastructure and services [47–50]. These approaches align with the goals of inclusive urban development by ensuring that all citizens, regardless of their physical abilities, can access public services equitably. Additionally, there is growing interest in the economic and social impacts of improved public transport accessibility for people with disabilities [19,34,51]. Furthermore, public transport contributes to climate-neutral urban policies by generating significantly lower emissions compared to private vehicles [52].

Physical disability is the most common type of disability in society and has therefore received more attention and research than other types. There is also agreater social awareness of this type, as physical barriers in public transport networks are more visible and apparent to the general population, such as the lack of ramps or lifts in underground stations or inaccessible buses for wheelchair users. It should be noted that, in some studies, people with physical disabilities, people with reduced mobility, and older adults are analysed together [53–55].

The type of disability influences the perceived barriers to using public transport; different disabilities create distinct barriers [19,37], although there are also commonalities. [36] found that people with visual and physical disabilities experienced a greater number of barriers than those with hearing impairments or other disability categories, highlighting the need for targeted interventions for these populations. The diversity of barriers requires a comprehensive and customised approach in the design of public transport accessibility policies and solutions [19,56].

Among transportation modes, urban buses are the most extensively researched, with questionnaires, semi-structured interviews, and focus groups being commonly employed tools [35,49,50,57–61]. However, few studies have been conducted through direct observation of the accessibility of public bus transport. Direct observation provides a first-hand account of accessibility challenges, capturing real-time issues that might be overlooked in questionnaires or interviews [62]. Studies highlight the importance of direct observation in identifying critical gaps in accessibility, and demonstrate the tangible benefits of investing in accessible infrastructure. [63–65]. Other studies have analysed bus stops from different perspectives, such as physical characteristics [66], pedestrian infrastructure [67, 68], impact on safety [69], or impact on travel behaviour [70], but often from the perspective of the general population rather than people with disabilities. Expanding these analyses to include accessibility issues would support the development of more inclusive and efficient transport systems.

Despite research on various aspects of public transport accessibility, there is a gap in studies focusing specifically on compliance with accessibility legislation, a notable deficiency in precise, real-time data on accessibility, and a lack of integration of the perspectives of people with disabilities into the design methodology. The participation of people with disabilities in the planning and design of transport is essential to adequately meet their mobility needs and improve user experience [46,48]. Understanding how the urban environment shapes the travel behaviour of people with disabilities is crucial for informing transport and urban planning decisions that promote their social integration [71–73]. Observational studies are often limited to specific infrastructures or sample areas and facilities [74,75]. These field observations are conducted using checklists that are not very comprehensive, with subjective evaluation criteria, and no reference to normative texts [63,71,76,77]. Addressing these gaps may contribute to improving transport systems and advancing towards more sustainable and inclusive urban mobility, in line with Sustainable Development Goals and urban planning frameworks.

The objective of this research paper is to evaluate the accessibility of bus stops in the city of Burgos (Spain) for people with disabilities and identify existing barriers and opportunities for improvement. This study not only assesses compliance with accessibility standards, but also integrates the lived experiences of users with disabilities, offering actionable insights for urban planners and policymakers. Burgos, the second most populous city in Castilla y León (the largest region in Europe), is a representative example of medium-sized cities with historic city centres and large industrial areas facing mobility challenges between different districts. Although research exists on different aspects of public transport accessibility, there are still gaps in studies that combine compliance with accessibility legislation with user-centred design methodologies. Through direct observation, this study identifies critical accessibility gaps and highlights the tangible benefits of investing in accessible infrastructure. These investments not only promote equity and inclusion in urban mobility for people with physical disabilities, but also benefit all people with reduced mobility, and more generally support sustainable urban development by encouraging a shift from private vehicles to public transport, thus promoting more inclusive and resilient cities. In addition, the replicability of this approach provides valuable insights for other urban contexts, even to promote urban mobility on a broader scale. The findings confirm that the methodological design effectively assesses accessibility conditions and provides a replicable framework to address them.

## Materials and methods

### Study area

**Fig 1:**
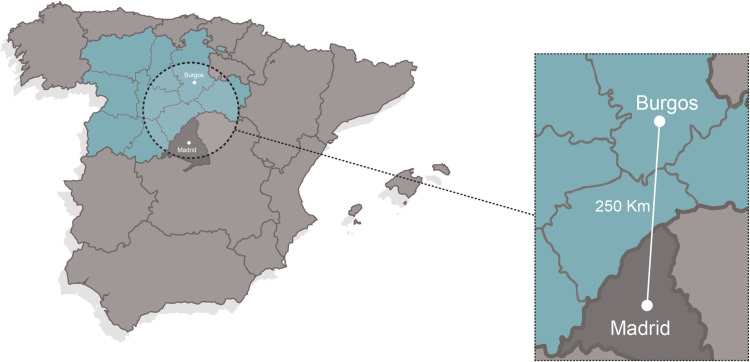
Location of Burgos on the map of Spain.

Burgos is a Spanish municipality located in the northern part of the Iberian Peninsula. It is the capital of the province of the same name and belongs to the autonomous community of Castile and Leon (highlighted in blue). The city is well-connected (approximately 250 km) to Madrid, Spain’s capital (Fig 1).

In 2023, Burgos had a population of 173,483 inhabitants [78], ranking as the 37th largest municipality in Spain and the second largest in Castile and Leon. The municipality of Burgos covers an area of 107.1 square kilometres and forms the core of a wider urban area that includes 31 surrounding municipalities. This combined area has a total population of 196,282 inhabitants [78]. The Burgos city bus network connects some of these municipalities to the city centre. The population distribution by age shows that 22.73% of the population is over 65. Additionally, there are 27,638 people with recognized disabilities in the province of Burgos, accounting for 7.8% of the population [79]. These data highlight the importance of addressing the mobility needs of this population.

Public transport by bus in Burgos is managed by the ‘Municipal Mobility and Transport Service’ [80]. The city’s bus network consists of 28 lines with 149 stops, including special services and night lines. The network covers the entire metropolitan area, extending approximately 21 km from the Cotar neighbourhood in the north to Arcos Road in the south, and 22 km between Villalonquejar in the west and Castanares in the east. In 2023, 13,296,914 passengers used the urban transport service, marking a 21.1% increase in ridership compared with 2022.

The Municipal Accessibility Plan of the City of Burgos [81] proposes accessibility improvements in four areas: communication, public roads, municipal buildings, and public transportation. Specifically, it defines 12 types of bus stops and allocates an estimated annual budget for the proposed improvements. These efforts are further supported by the Sustainable Urban Mobility Plan (PMUS) approved in 2021, which emphasizes safe, equitable, and environmentally friendly transportation [82]. The PMUS underscores the importance of making public transport stops and stations accessible by establishing design criteria for new bus shelters, adapting signage, and modifying existing infrastructure to ensure compliance with the Accessibility Plan.

Aligned with SDG 11, the PMUS promotes a sustainable urban environment that prioritizes accessibility for vulnerable groups, including people with disabilities and older adults. It identifies improvements in public transport infrastructure as essential to reduce car dependency, lower emissions, and improve social inclusion. This approach incorporates universal design principles across all facilities, and includes continuous monitoring to ensure that accessibility meets the community’s needs, ultimately supporting a more inclusive and resilient city.

### Data collection and methodology

This study applies, reviews, and enhances the methodology proposed by [83] to evaluate the accessibility of urban bus stops in Burgos. Conducted between January and June 2022, the research validates the methodology’s replicability and adaptability to other urban contexts. The objective is to evaluate public transport accessibility for people with physical disabilities and/or reduced mobility, with a focus on user autonomy and safety. The methodology integrates both legal parameters established in relevant regulations and legislation, as well as the lived experiences and perspectives of people with disabilities. This dual approach provides a more comprehensive understanding of accessibility, moving beyond a strictly legal perspective.

To ensure a holistic and user-centred approach, the authors collaborated with COCEMFE, a non-profit organisation representing 71 member entities that advocates for the rights of individuals with physical and organic disabilities. COCEMFE’s involvement was instrumental in grounding the evaluation process in the real experiences and challenges faced by people with disabilities, ensuring that the methodology went beyond theoretical frameworks.

The first step in the process was to draw up an initial list of requirements, based on an in-depth analysis of current regulations and legislation on accessibility in public transport, in close collaboration with COCEMFE technicians (Table 1).

**Table 1:** COCEMFE staff collaborating in the process.

Person	Academic Degree	Position at COCEMFE	Professional experience related to accessibility
M.A.E	Clinical Psychology	Coordinator	Coordination and management of various areas of the organization. Design and management of universal accessibility projects
M.A.D	Technical Architect	Accessibility Consultant	Universal accessibility consultant. Accessibility advice for people with disabilities and families. Participation in accessibility projects
C.C.V	Technical Architect	Accessibility Consultant	Universal accessibility consultant. Accessibility advice for people with disabilities and families. Participation in accessibility projects
R.Z	Technical Architect	Accessibility Consultant	Universal accessibility consultant. Accessibility advice for people with disabilities and families. Participation in accessibility projects
R.M.G.	Social Education	Social Educator/ Volunteer Coordinator	Regular user of the COCEMFE Accessibility application. Person with physical disability

During this process, references were made to primary legal texts, including any modifications and/or amendments. The most relevant document in Spain is Royal Decree 1544/2007 [84], which regulates the basic conditions of accessibility and non-discrimination for people with disabilities in accessing and using transportation. The annexes of this decree outline accessibility requirements for various transport modes. During the analysis meetings, additional requirements not specified in normative texts, which can significantly limit access to public transport for individuals with physical disabilities, were also identified.

The initial list of requirements was verified in two focus groups with COCEMFE users with physical disabilities, whose characteristics are presented in Table 2. The participants were selected by COCEMFE to ensure diversity in terms of gender, age, place of origin, and degree of disability. These focus group sessions were conducted in person to ensure that the criteria were relevant from their perspective. These focus groups aimed to confirm that the selected criteria accurately reflected users’ needs, ensuring that the evaluation process was both inclusive and practical.

**Table 2:** People with disabilities participating in focus groups.

Person	Gender	Age	Degree of disability
A.M.C.	Female	43	53%
B.C.Y.	Female	37	82%
B.I.M.	Male	35	62%
C.D.T.	Male	31	64%
G.E.B.	Male	46	67%
I.M.O.	Male	29	75%
R.F.D.	Male	38	71%
R.M.G.	Male	23	78%
S.H.P.	Male	21	65%
A.M.C.	Male	43	53%

During the focus group discussions conducted in January 2022, it became evident that some of the initially identified requirements were overly stringent, requiring adjustments to their benchmarks. However, certain requirements, while not critical for individuals with physical disabilities, were deemed essential for people with other disabilities, such as those with visual or hearing impairments. Consequently, these requirements were retained to ensure a more inclusive approach.

After reaching a consensus in the focus groups, the requirements were categorised as critical or non-critical based on the participants’ opinions. Critical requirements must always be met to ensure the autonomous and safe use of public transport by people with physical disabilities or reduced mobility. Examples include ramps, sufficient pavement width, and proximity to lowered kerbs. Non-critical requirements, while important according to regulations, can be managed by people with physical disabilities without significantly affecting their autonomy and safety when using public transport. Examples include the availability of information in accessible formats such as Braille or audio.

The classification of requirements was guided by the question: “Does the absence of this requirement impact your ability to use public transport autonomously and safely?”

As a result, a total of 12 critical requirements and 11 non-critical requirements were approved, as detailed in Table 3. Each requirement, whether critical or non-critical, was further categorised based on its position within the accessibility chain, distinguishing whether it pertained to access to the bus stop or the stop itself [85]. To facilitate the evaluation process, each requirement was assigned a unique identifier according to its category: CA (Critical Access to Stop), CS (Critical Stop), NCA (Non-Critical Access to Stop), and NCS (Non-Critical Stop).

**Table 3:** Requirements analysed Bus.

Type	Id.	Requirement
Critical Access to stop	CA.1	Unevenness saved by ramps (no steps)
	CA.2	Pavements without parts or loose items, no sliding, solid and continuous
	CA.3	Sidewalk wide enough
	CA.4	Wide enough pavement
	CA.5	Existence of step free access road crossing at a distance less than 100 m
Critical Stop	CS.1	Traffic protection at the start/ end in the bus stop, preventing other vehicles stopping and blocking access.
	CS.2	Existence of bus shelter
	CS.3	Bus shelter side/ centre, min step access. 0.90 m
	CS.4	Bus shelter clear width 1.50 m to 25 cm in height and 1.35 m to 2.10 m and 2.10 m headroom
	CS.5	Appropriate and clear identification of transparent or translucent elements present: signalled with 2 horizontal stripes of 5–10 cm in width and at a height of between 0.70 to 0.80 m and 1.40–1.70 m from floor level and made of brightly coloured material
	CS.6	Clearance height of 2,10 metres
	CS.7	At least 1 ischiatic support and 1 standard seat
Noncritical access to stop	NCA.1	Gratings and manhole cover flush with flooring
	NCA.2	Tree trenches covered or flush with pavement
Noncritical stop	NCS.1	Tactile-visual pavement stripe 1.20 metres wide, perpendicular to the direction of travel and from curb to facade, with a minimum visual touch of 40 cm at the curb
	NCS.2	Characters Line Identification height 14 cm contrasting colour
	NCS.3	Signpost with bus stop number and routes served
	NCS.4	Line information: Identification and denomination in Braille on the signpost
	NCS.5	Line Information: Identification, name and route in Braille on the marquee bus shelter
	NCS.6	Seats with armrests at the end of bus shelter
	NCS.7	0.45 cm seat height clearance from ground +/- 2 cm in the shelter
	NCS.8	Digital display information panel
	NCS.9	Digital display with audible information available

The developed checklist, provided in S1 Appendix, was validated through direct observation and completion at bus stops across Castile and León. This validation process was conducted in collaboration with COCEMFE technicians and users with physical disabilities to ensure its practicality and relevance. Data were systematically collected using the validated checklist, which was designed to be clear, user-friendly, and free of overly technical language.

The authors, together with COCEMFE staff, conducted the fieldwork, with individuals with physical disabilities participating at selected stops. A systematic procedure ensured a thorough and accurate accessibility assessment of all 431 bus stops in Burgos. Initially, two individuals performed direct observations at each stop, evaluating both critical and non-critical requirements identified in the study methodology. Each stop was examined for approximately 15 minutes, with every requirement assessed and recorded in the checklist. Responses were marked as ‘yes’, ‘no’, or ‘not applicable’, depending on the situation. Detailed observations and additional notes documented any relevant information.

To ensure accurate identification of each stop for future reference, GPS coordinates were used to geolocate each bus stop. Additionally, photographs (taken by the authors) documented the condition and environment of each stop at the time of assessment. These photographs provided a visual record of the physical condition of the stops, highlighting barriers that may not have been immediately apparent from the written records. Furthermore, the images served as a reference point for subsequent discussions and evaluations, ensuring a consistent understanding of each stop’s condition among all team members.

The collected data were organised and analysed using specifically designed templates in Microsoft Excel. These templates enabled clear and orderly recording of the results, along with automated calculations and graphical representations.

The methodology is fully replicable in other urban contexts, as it is based on a structured and standardised checklist. The evaluation process consists of applying the checklist through on-site assessments, complemented by photographic documentation and geolocation of the stops, followed by tabulation and analysis of the results. The checklist used in this study is provided as supplementary material (S1 Appendix), facilitating its application in future research. While the core methodology remains unchanged, minor adaptations may be required to align with the specific accessibility regulations of each country.

### Ethic statement

This study involved human participants who voluntarily participated in focus groups to ensure that the criteria for evaluating public transport accessibility were relevant from their perspective. Participants were recruited and participated in the study between 1 January 2022 and 30 June 2022. They were fully informed about the purpose of the study, how their contributions would be used, and their right to withdraw at any time without consequences. All participants were adults; no minors were involved in the study.

Oral informed consent was obtained prior to participation, and all personal data were fully anonymised to ensure privacy and confidentiality. Under no circumstances can these data be used to identify individuals.

This study did not require formal ethical approval from an institutional review board (IRB) or ethics committee because it did not involve sensitive personal data, clinical interventions, or experiments. The study adhered to ethical guidelines for social research, respecting participants’ rights and dignity throughout the process.

## Results

A significant number of bus stops are concentrated in the city centre of Burgos. However, variations in the design, location, distribution, and equipment of these stops exist depending on the type of area (e.g., old town, suburbs, industrial zones, and nearby towns). A total of 431 bus stops in Burgos were assessed using in-situ observations. Of these, 26 lacked any form of identification, such as shelters, signage, or information displays, meaning that not all accessibility requirements could be applied. Nevertheless, these stops were not entirely excluded from the analysis; instead, the presence of a line post and/or a shelter was assessed, with both elements classified as ‘No’. For the remaining 405 stops, all requirements were reviewed.

### Critical requirements

**Fig 2:**
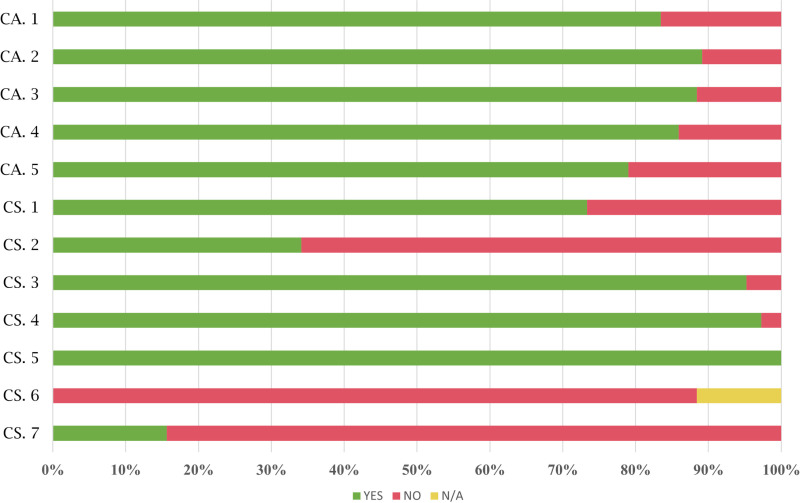
Results for critical requirements at bus stops.

Fig 2 shows the results for each of the critical requirements for both access to the stop (CA) and the stop itself (CS).

**Fig 3:**
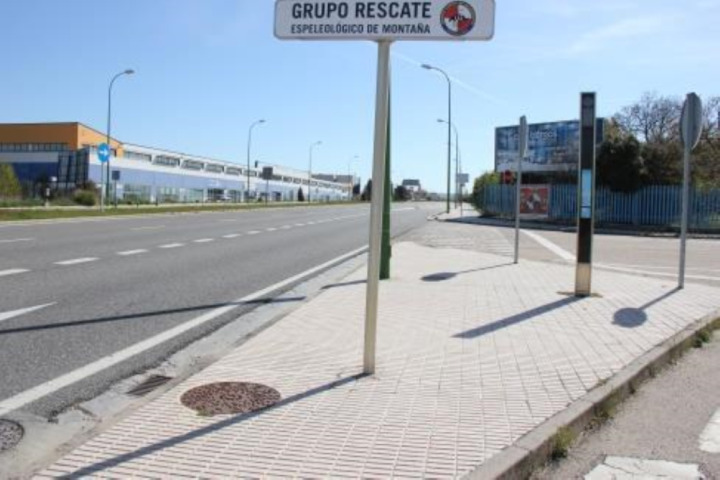
Inaccessible bus stop.

68.91% of the bus stops meet all critical requirements for access to the bus stop (CA). This percentage increases to 73.33% when excluding bus stops that were not analysed because they lacked identification. The infrastructure of the city of Burgos and nearby municipalities generally allows access to bus stops, although some of them are very difficult to access for people with physical disabilities, such as the bus stop shown in Fig 3.

The route to the bus stop must be accessible and easy to use for everyone. Slopes must be bridged by ramps or fords to allow wheelchair users to move around independently (CA1). 83.46% of bus stops have ramps and step-free access, allowing individuals with physical disabilities to access the stop independently.

**Fig 4:**
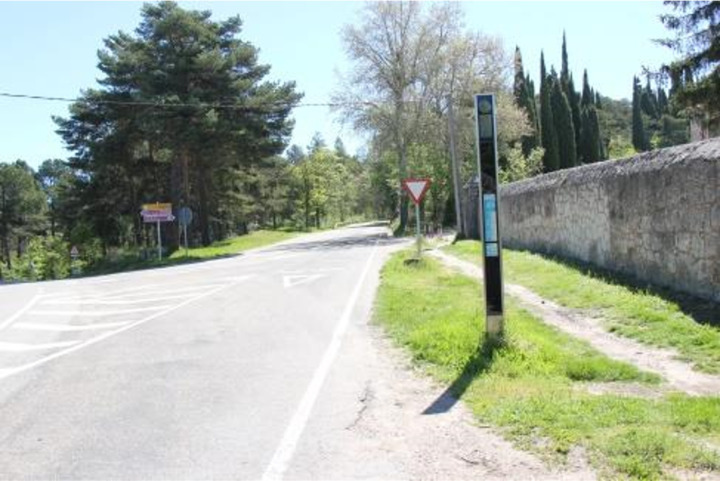
Bus stop with unsuitable pavement.

The pavement is an essential element for the accessibility of urban itineraries. It needs to be strong and uniform to withstand the movement and dragging of certain elements, such as a wheelchair (CA2). Irrespective of the type of flooring used, with different sizes, colours, and materials, most stops (88.80%) have non-slip, continuous, and compact flooring. On the other hand, the rest of the stops have surfaces that make it difficult for people with physical disabilities to move around and can cause them to fall. These stops, such as the one shown in Fig 4, are usually located on the periphery (where there are no pavements) or in industrial estates.

**Fig 5:**
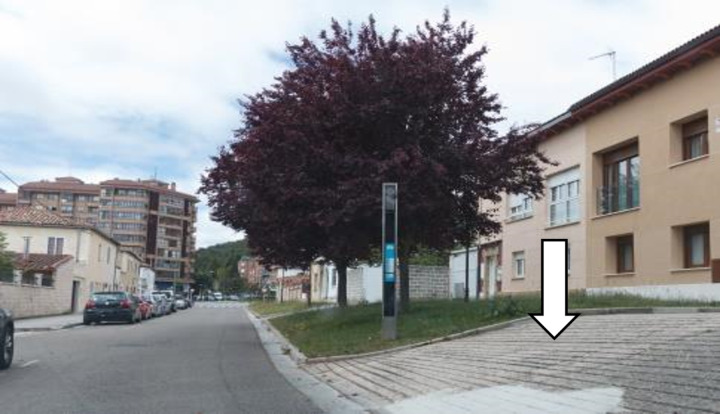
Bus stop with a steep slope.

The sidewalk slope (CA3) is satisfactory at 88.40% of the stops. However, 44 stops exceed the established values (less than or equal to 4% in the direction of travel and 2% in the transverse direction). These stops are primarily located in the upper old town. The slope is particularly important for people with physical disabilities to move around easily and without excessive effort. An example of a bus stop that does not meet this requirement is shown in Fig 5.

The pavement width is a crucial factor in ensuring the accessibility and safety of urban routes. Pavements must be at least 1.50 metres wide (CA4) to facilitate passage for wheelchair users. However, a significant number of bus stops (14.07%) have very narrow pavements that impede the passage of people with disabilities. These bus stops are mostly found in the older areas of the city where pavements tend to be narrower.

**Fig 6:**
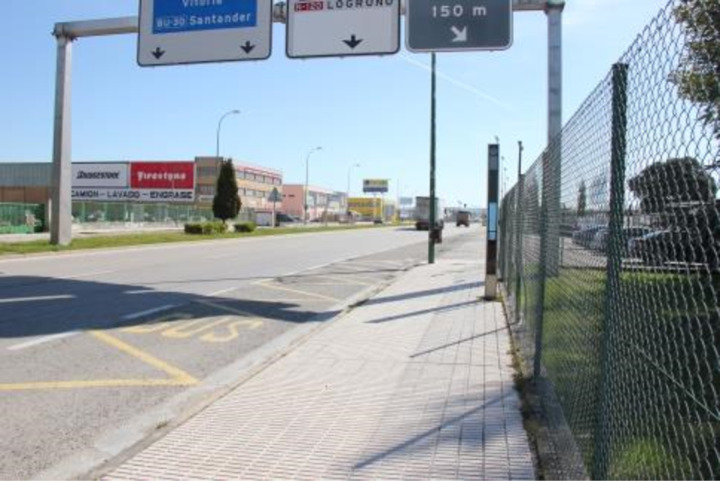
Bus stop with step-free access located over 100 metres away.

The distance from a bus stop to a lowered kerb is critical for a person with a physical disability to access the stop comfortably and safely. Focus groups estimated that 100 metres is a reasonable distance (CA5). 79.01% of the stops meet this requirement, while 85 stops do not, primarily in industrial estates (Fig 6).

Regarding the critical stop requirements (CS), at the time of the study, none of the 431 bus stops met all seven requirements. One critical requirement for people with physical disabilities is the protection of the stop with rigid elements to prevent vehicle encroachment (CS1). A rigid element is a kerb extension or another fixed element that cannot be moved by a person to park in that space, such as a traffic cone or bollard. If this space is occupied or obstructed, the bus will not be able to approach the pavement or deploy the wheelchair ramp.

**Fig 7:**
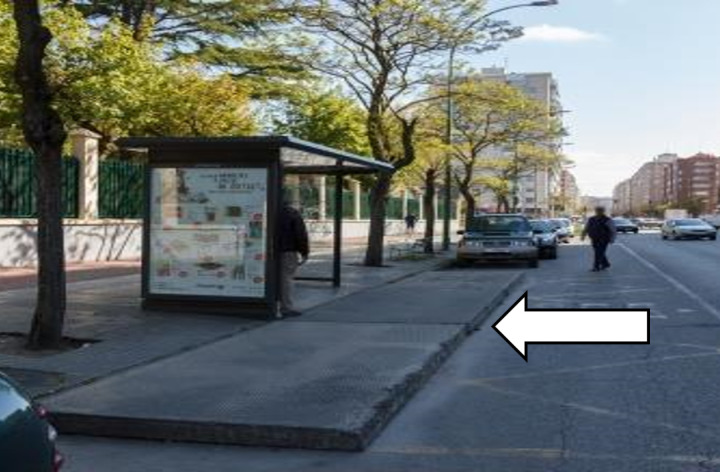
Protection at bus stops. Platform extending from the sidewalk.

**Fig 8:**
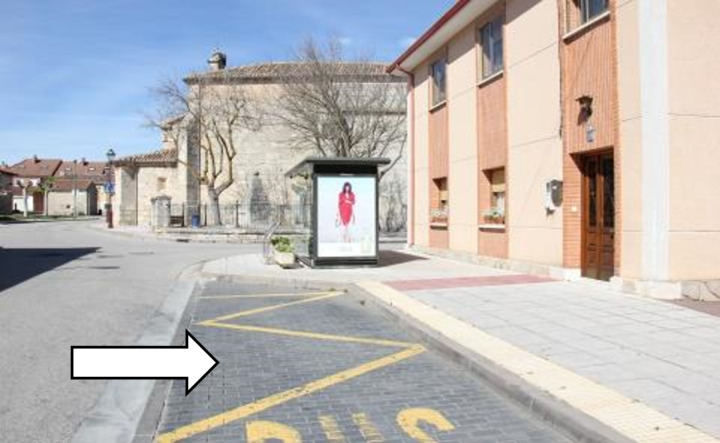
Protection at bus stops. Road markings indicating bus stop.

In Burgos, no bus stop has this type of protection. However, in many cases, as shown in Fig 7, a platform has been built from the pavement to the traffic lane to ensure a good approach for the bus to the stop. In other cases (Fig 8), a dedicated lane has been designed, or the area has been marked with road markings.

**Fig 9:**
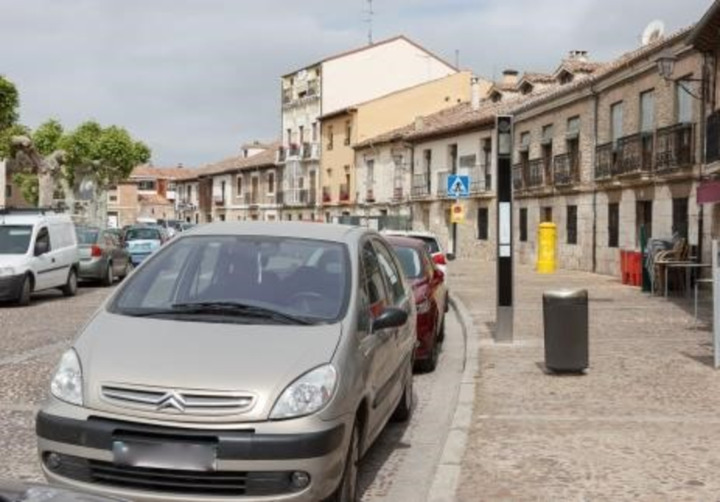
Vehicles obstructing bus access.

We consider that 73.33% of bus stops provide a suitable approach for disabled passengers to get on and off the bus. Issues include on-road stop locations, short platforms that hinder bus proximity, and parked vehicles obstructing the designated zone. Fig 9 illustrates how parked cars can make it impossible or dangerous for individuals to access the bus. Additionally, designated parking spaces are often inadequate for large, articulated buses.

**Fig 10:**
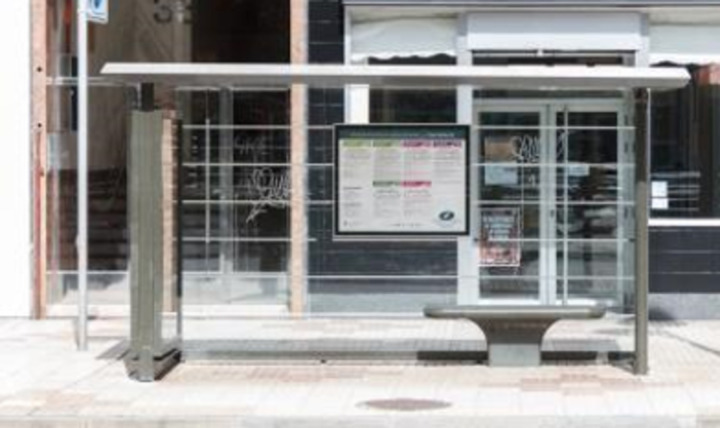
Bus shelter installed in Burgos.

The presence of a bus shelter (CS2) at each stop is not a legislative requirement, but it does make waiting for passengers more comfortable and protects them from weather. If a stop has a bus shelter, it must meet specific requirements. The format of the bus shelters installed in Burgos is shown in Fig 10.

**Fig 11:**
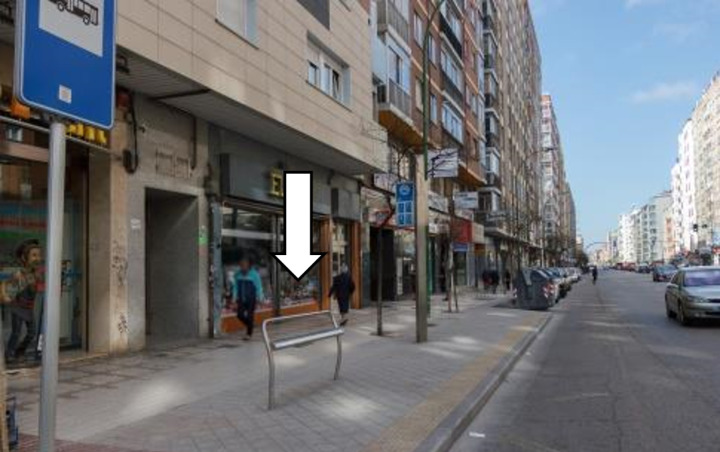
Bus shelter installed in Burgos.

As of the date of the study, 34.11% of the stops had a bus shelter, and the degree of compliance with the requirements is very high for access (CS3) and minimum dimensions in width (CS4) and height (CS6). All bus shelters have seats, and 15.65% have ischial supports (CS7) to make it easier for a person on crutches with stability issues to sit while waiting for the bus. Ischial supports are also being installed at stops without bus shelters, such as the one in Fig 11.

None of the installed bus shelters meet the requirement for ‘transparent enclosure signage’ (CS5). This requirement involves the presence of two horizontal bands in a contrasting colour to clearly differentiate the glass enclosure and prevent accidental collisions. The current bus shelters have five horizontal bands of smaller width than required.

### Non-critical Requirements

**Fig 12:**
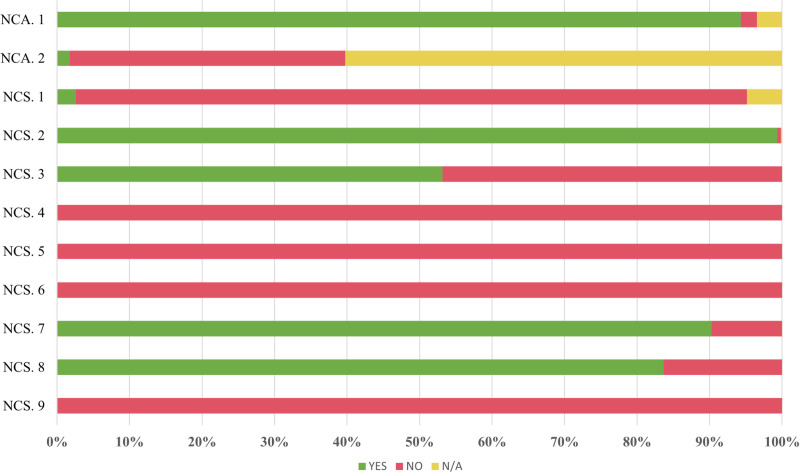
Results for non-critical requirements at bus stops.

Non-critical requirements, although not imperative for safety and autonomy, significantly enhance the user experience. Fig 12 shows the results for each of the non-critical requirements.

**Fig 13:**
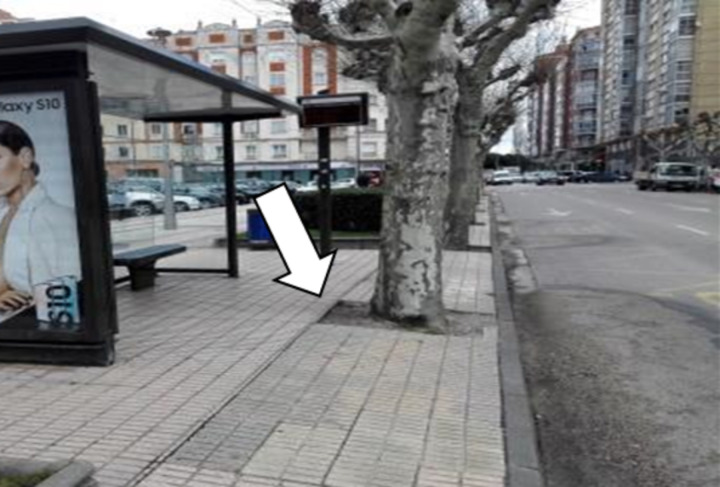
Tree pits not flush with the pavement.

Manhole covers, grilles, gratings, and tree surrounds can be major obstacles for people with reduced mobility, as they can cause trips and falls. It is important that they are flush with the pavement (NCA1-NCA2), although it is possible to avoid driving over them if there is sufficient space. 94.32% of the stops have manhole covers, grilles, and gratings that are flush with the pavement. However, a significant percentage of tree surrounds (38.2%), as shown in Fig 13, are either not covered or not flush with the pavement.

**Fig 14:**
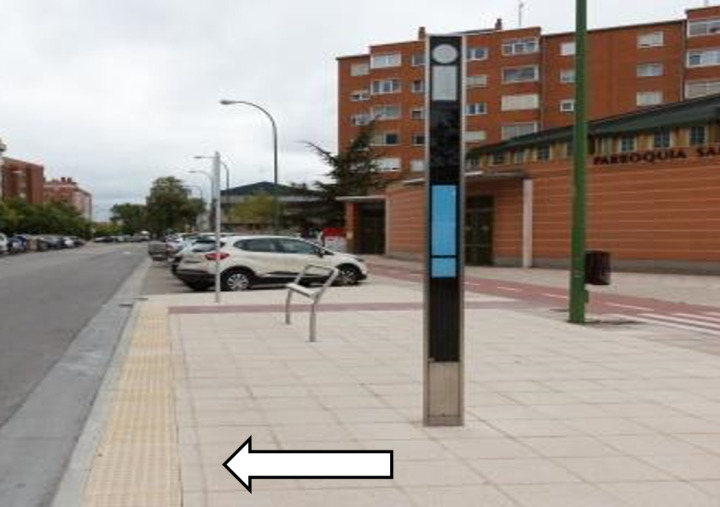
Tactile-visual pavement stripe.

The tactile-visual paving strip allows visually impaired individuals to detect the presence of a bus stop and orient themselves towards it. This element, shown in Fig 14, is characterised by a textured surface with slight reliefs and a colour that contrasts with the rest of the pavement. 88.54% of bus stops do not have the required 1.20-metre-wide tactile-visual paving strip perpendicular to the direction of travel (NCS1), as mandated by current regulations. However, this is a non-critical requirement for people with physical disabilities.

**Fig 15:**
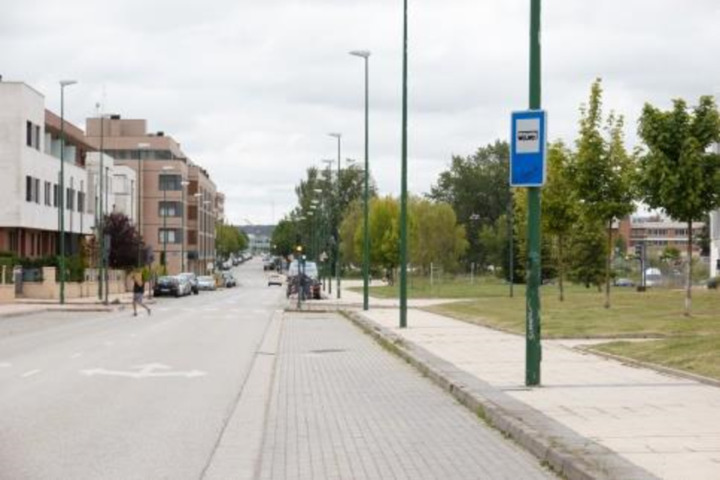
Stop lacking line identification on bus pole.

The latest requirements analysed refer to the existence of a signpost line (NCS3), bus shelter, and bus information display. If these elements are present, they must also meet other requirements such as information in Braille on the signpost or a sound device for the bus information display. Information at stops is totally inadequate. Line identification is absent at many stops, such as the one shown in Fig 15, where there is neither a signpost nor a bus shelter.

At stops where these elements are present, the contrast and size of letters or numbers indicating the line are often insufficient for easy identification (NCS2). The location and identification of routes on existing maps are also very difficult to understand. None of the signposts and bus shelters provide information, names, or routes of the lines in Braille (NCS4-NCS5).

**Fig 16:**
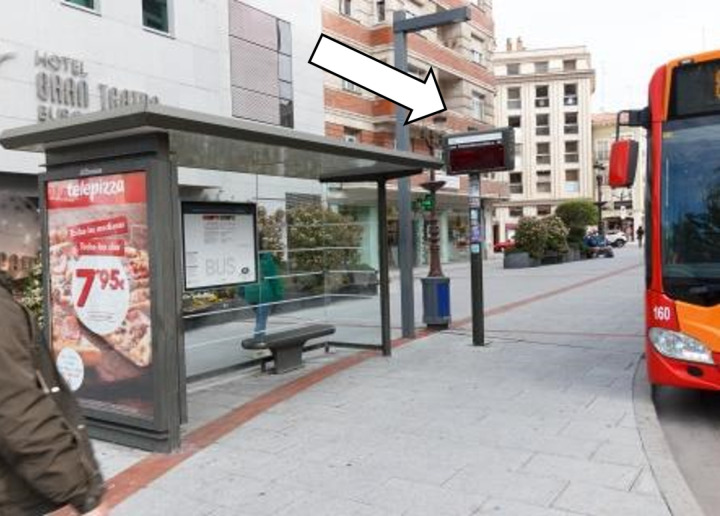
Stop with an information display.

Nine stops (5.81%) have at least armrests on their outboard seats (NCS6). The seat height requirement (NCS7) is met in the majority of cases (98.71%). Sixty-seven bus stops (15.55%) are equipped with bus information displays (NCS8) like the one in Fig 16. None of these stops have an audible announcement system (NCS9) for the visually impaired, nor a control or alternative system for the visually impaired.

## Discussion

Despite significant advances in the adaptation of public transport in Burgos following the approval of Royal Decree 1544/2007 [84], not all accessibility requirements established in current regulations are being met, nor are the additional criteria identified as necessary for people with physical disabilities to use public transport independently and safely. These findings align with previous studies on public transport accessibility, which have observed that, while some cities have implemented improvements, significant barriers to independent access for people with disabilities persist [3,19,36,56,86]. This highlights the global challenge of translating legislative advancements into practical and effectively applied solutions in urban environments. Continuous efforts to renovate bus stops and shelters demonstrate the city’s commitment to enhancing accessibility, but more comprehensive measures are still required to fully meet user needs.

Our study reveals persistent barriers to bus stop accessibility in Burgos, reflecting two core challenges in urban accessibility: structural limitations tied to historical or geographic constraints, and deficiencies in facilities that hinder inclusive design implementation. These findings align with previous studies [83,86], which also identify the interplay between physical barriers and inadequate infrastructure as significant obstacles to achieving urban mobility equity.

While general access to bus stops is good, with wide and well-maintained pavements and mostly accessible routes, a notable percentage of stops are inaccessible for various reasons:

- Located in areas of the city where pavements are narrower.
- Located in the upper part of the old town, where the streets are steeper.
- Located in areas where there are intermediate gardens between the pedestrian route and the bus stop.
- Located in industrial areas and on routes that connect the city with neighbourhoods far from the city centre.

Although certain accessibility barriers may be more frequently observed in specific areas of the city, such as steeper streets in the historic centre or narrower pavements in some neighbourhoods, there is no clear and uniform pattern linking accessibility deficiencies to particular urban zones. The presence or absence of such barriers depends on multiple factors, including historical urban layouts, urban renovation policies, and localised infrastructural modifications.

The compliance with critical accessibility requirements at bus stops has a considerable impact on the overall accessibility of the city. The following key actions are necessary to enhance inclusivity of people with physical disabilities or reduced mobility:

- Install protections at the start and end of bus stops to prevent encroachment by other vehicles. Current obstructions often prevent buses from parking correctly, making access difficult or even impossible for people with physical disabilities.
- Ensure that transparent glass enclosures of bus shelters meet the signage requirements established by current regulations, helping visually impaired individuals avoid collisions by clearly distinguishing the glass walls. While some shelters already include signage, they do not meet the required standard of two horizontal lines.

Non-critical requirements are not fully met at any bus stop. While these requirements are not essential for a person with a physical disability to access or remain at the bus stop, these features significantly impact the experience of individuals with visual or hearing impairments. Key areas for improvement include:

- Enhancing the availability of information: Many stops lack line identification. Additionally, existing maps and line numbers often fail to meet the contrast and size requirements (14 cm), making them difficult to read.
- Adding Braille information: No Braille information is available on signposts or bus shelters, limiting accessibility for visually impaired users.
- Providing armrests on bus shelter seats: Armrests, which provide essential support for individuals with mobility impairments, are absent in most shelters.
- Implementing audible information systems: There are no audible information displays at bus stops, which are essential for visually impaired users.

Addressing these interconnected issues is crucial to create a more accessible and equitable public transport system for all users, improving mobility and inclusivity.

The primary limitation of this study is the time elapsed between the fieldwork, data tabulation, analysis, and writing, during which certain changes in the city’s infrastructure may have slightly altered the findings. This limitation underscores the importance of continuous monitoring of urban accessibility to maintain relevance and applicability over time. Nevertheless, the developed methodology remains highly adaptable and can be applied to other cities facing similar challenges. This transferability highlights its potential as a practical framework for cities seeking to address barriers in public transportation systems and to advance sustainable urban mobility.

Future research will extend this work to other cities, including evaluations of major train stations, enabling more comprehensive and cross-modal comparisons. Additionally, it could investigate accessibility challenges faced by individuals with sensory or cognitive impairments, thereby broadening the concept of mobility as a fundamental human right. This research could also assess recent infrastructure developments and, through longitudinal studies, evaluate the effectiveness of implemented interventions over time. Furthermore, future studies could develop a methodology for prioritising investments in bus stops, providing policymakers with a structured framework to optimise resource allocation. Such an approach would facilitate the identification of the most impactful interventions, ensuring that investments effectively enhance the mobility of people with disabilities.

This user-centred methodology represents a significant contribution to addressing accessibility challenges in urban mobility systems globally. It aligns with the growing demand for inclusive and sustainable transport solutions, as reflected in Sustainable Development Goals and urban planning frameworks. As highlighted in [86], comparative studies play a crucial role in monitoring progress, identifying best practices, and adapting strategies to diverse urban contexts. By adopting this approach, future research can provide valuable insights into the effectiveness of accessibility interventions, promoting the widespread adoption of effective strategies. These efforts will drive the development of public transport systems that equitably benefit all citizens. By overcoming accessibility challenges, cities can contribute to more cohesive and equitable urban environments while advancing the inclusive and resilient urban mobility envisioned by global frameworks such as the Sustainable Development Goals.

## Conclusions

This study evaluates the accessibility of bus stops in the city of Burgos, considering not only compliance with current regulations but also the ability for independent and safe use of public transport by people with physical disabilities or reduced mobility. By adopting a user-centered methodology developed by [83], this research advances a holistic framework for assessing accessibility that not only integrates physical infrastructure analysis but also incorporates the lived experiences of users. Tested and validated in other Spanish cities [83–86], it demonstrates adaptability and effectiveness across diverse urban contexts, making it a valuable tool for application in any city. Consistent with earlier research, we emphasize the critical role of evaluation in shaping effective and sustainable mobility policies [87].

The results of this study have significant practical implications for transportation policy formulation and urban planning, not only in Burgos but also in other cities facing similar challenges. By applying this methodology, policymakers can identify key areas for improvement and prioritize interventions, contributing to more inclusive and accessible public transport systems. Such targeted actions are essential to address the pressing need for equitable urban mobility. The results of this study highlight the need to integrate accessibility considerations into urban and transport planning processes from design, ensuring that the identified barriers are systematically addressed to improve the overall inclusiveness and efficiency of public transport systems.

Furthermore, the active involvement of individuals with disabilities in the assessment process not only enriched the findings but also ensured that the recommendations are grounded in lived experiences. Engaging these stakeholders in ongoing planning and decision-making processes is essential for developing truly inclusive urban environments. This participatory approach enhances the legitimacy and relevance of the proposed solutions, fostering greater collaboration between public authorities and civil society.

The implementation of the recommendations derived from this study can significantly enhance the accessibility and usability of Burgos’s public transport system, promoting greater inclusion and equity for all users. These actions will not only benefit people with disabilities but also foster a more sustainable urban environment for the entire community. By reducing reliance on private vehicles, alleviating traffic congestion, and decreasing emissions of harmful pollutants, improved public transport accessibility supports the transition towards a more efficient and environmentally friendly transport system. This dual benefit of inclusivity and sustainability positions accessibility as a cornerstone of modern urban development strategies. This approach aligns with the United Nations Sustainable Development Goals (SDGs), particularly SDG 11, which advocates for inclusive and sustainable cities. By prioritizing inclusive transport infrastructure, cities such as Burgos can lead the way in demonstrating how urban environments can be made equitable and sustainable for all residents.

Although the study has focused on Burgos, the developed methodology is highly replicable and adaptable to other cities facing similar challenges. This approach can assist other municipalities in identifying priority areas for intervention, fostering a more inclusive and accessible public transport system for all citizens. By embracing these recommendations, cities can ensure that public transport systems not only meet regulatory standards but genuinely serve the needs of all residents, paving the way for more inclusive and resilient urban environments.

Ultimately, this work contributes to the broader discourse on urban accessibility and sustainable development, offering valuable insights for urban planners, policymakers, and disability advocates globally. This study, alongside previous research [83,87], supports associations of people with disabilities in advocating for the enforcement of accessibility laws and regulations by public administrations. By shedding light on the barriers to public transport accessibility, these findings collectively raise awareness of the issue and promote greater accountability among policymakers, urban planners, and society.

## Supporting information

S1 Appendix Accessibility assessment checklist for public bus transport. This appendix contains the complete checklist used to evaluate the accessibility of bus stops in Burgos, detailing critical and non-critical requirements.
(PDF)
