# Supplementary material for: Assessing public transport accessibility for people with physical disabilities in burgos, spain: A user-centered approach to inclusive urban mobility
Source: PLoS One. 2025 Apr 29;20(4):e0322068. doi: 10.1371/journal.pone.0322068 (PMC12040260; doi:10.1371/journal.pone.0322068)
Supplement: S1 Appendix — This appendix contains the complete checklist used to evaluate the accessibility of bus stops in Burgos, detailing critical and non-critical requirements. (PDF) [file pone.0322068.s001.pdf]

ACCESSIBILITY ASSESSMENT FORM FOR PUBLIC BUS TRANSPORT

BUS STOP IDENTIFICATION		PHOTOGRAPH
BUS ROUTE(S)	BUS STOP ADDRESS	
FORM NUMBER	BUS STOP IDENTIFICATION CODE	

ANALYSIS OF CRITICAL REQUIREMENTS

TYPE	ID.	REQUIREMENT	YES	NO	N/A	NOTES
Access to Stop	CA.1	Unevenness saved by ramps (no steps)				
	CA.2	Pavements without parts or loose items, no sliding, solid and continuous				
	CA.3	Sidewalk wide enough				
	CA.4	Wide enough pavement				
	CA.5	Existence of step free access road crossing at a distance less than 100 m				
Stop	CS.1	Traffic protection at the start / end in the bus stop, preventing other vehicles stopping and blocking access.				
	CS.2	Existence of bus shelter				
	CS.3	Bus shelter side / centre, min step access. 0.90 m				
	CS.4	Bus shelter clear width 1.50 m to 25 cm in height and 1.35 m to 2.10 m and 2.10 m headroom				
	CS.5	Appropriate and clear identification of transparent or translucent elements present: signalled with 2 horizontal stripes of 5-10 cm in width and at a height of between 0.70 to 0.80 m and 1.40-1.70 m from floor level and made of brightly coloured material				
	CS.6	Clearance height of 2,10 metres				
	CS.7	At least 1 ischiatic support and 1 standard seat				

ANALYSIS OF NON-CRITICAL REQUIREMENTS

TYPE	ID.	REQUIREMENT	YES	NO	N/A	NOTES
Access to Stop	NCA.1	Gratings and manhole covers flush with flooring				
	NCA.2	Tree trenches covered or flush with pavement				
Stop	NCS.1	Tactile-visual pavement stripe 1.20 metres wide, perpendicular to the direction of travel and from curb to facade, with a minimum visual touch of 40 cm at the curb				
	NCS.2	Characters Line Identification height 14 cm contrasting colour				
	NCS.3	Signpost with bus stop number and routes served				
	NCS.4	Line information: Identification and denomination in Braille on the signpost				
	NCS.5	Line Information: Identification, name and route in Braille on the marquee bus shelter				
	NCS.6	Seats with armrests at the end of bus shelter				
	NCS.7	0.45 cm seat height clearance from ground +/- 2cm in the shelter				
	NCS.8	Digital display information panel				
	NCS.9	Digital display with audible information available				

ADDITIONAL REMARKS
